# How to demix Alzheimer-type and PSP-type tau lesions out of their mixture -hybrid approach to dissect comorbidity-

**DOI:** 10.1186/s40478-019-0708-4

**Published:** 2019-05-06

**Authors:** Momoko Ebashi, Yoshinori Ito, Miho Uematsu, Ayako Nakamura, Katsuiku Hirokawa, Satoshi Kamei, Toshiki Uchihara

**Affiliations:** 1 Laboratory of Structural Neuropathology, Tokyo Metropolitan Institute of Medical Science, Tokyo, Japan; 2 Department of Neurology, Nihon University, School of Medicine, Tokyo, Japan; 3 Department of Neurology, Yokufukai Hospital, Tokyo, Japan; 4 Department of Pathology, Nitobe-Memorial Nakano General Hospital, Tokyo, Japan; 5 Neurology Clinic with Neuromorphomics laboratory, Nitobe-Memorial Nakano General Hospital, Tokyo, Japan

**Keywords:** Tau cytopathogy, AD, PSP, Comorbiditiy, Isoform, Glia

## Abstract

Neurofibrillary tangles (NFTs), are shared between progressive supranuclear palsy (PSP) and Alzheimer disease (AD). Histological distinction of PSP and AD is possible based on the distribution of NFTs. However, neuropathologists may encounter diagnostic difficulty with comorbidity of PSP and AD. In this study, we tried to circumvent this difficulty by analyzing five autopsied brains harboring both PSP and AD pathology. Tau-positive lesions were sorted based on their cell type (neuron versus glia), and tau isoforms: three-repeat (3R) versus four-repeat (4R) tau. 16 regions were selected to map these lesions throughout the brain. 4R-tau lesions were present in all areas examined. Among them, 3R-tau lesions were absent in some areas. These 4R selective (4R+/3R-) areas dictate prototypic distribution of PSP, not usually found in AD, such as pontine nucleus, red nucleus, inferior olivary nucleus, dentate nucleus, globus pallidus and putamen, each contained both glial and neuronal lesions. In contrast, additional 3R-tau lesions were found in hippocampal formation to neocortex, where 3R immunoreactivity (IR) was predominant over the 4R counterpart mainly in neurons as found in AD but not in PSP. Although tau lesions in central grey matter, substantia nigra and locus coeruleus are found in both AD and PSP, 4R-selectivity with glial component suggests PSP origin. Even if the presence of 3 R IR in these areas suggests AD pathology, it does not exclude the involvement of PSP-type lesion because distinction of 4R IR into PSP or AD is not yet possible. Further demixing may be possible if biochemical difference of 4R tau between PSP and AD is identified.

## Introduction

Alzheimer disease (AD) and progressive supranuclear palsy (PSP) are characterized by deposition of tau in the brain. Initially, histological definitions were based on the disease-specific distribution of argyrophilic neurofibrillary tangles (NFTs), which are quite distinct between AD [1] and PSP [31]. Another histological hallmark of PSP is tuft-shaped astrocytes (TAs) [15], which are essentially not found in pure AD. Because clinicopathological spectra of PSP and of AD are still expanding [10, 30], accurate clinical diagnosis is more and more complex and difficult. This diagnostic difficulty is much enhanced in aged population, where cormorbid pathologies, such as AD or Lewy pathology, may be encountered. [10, 30]. Such comorbidity is challenging to neuropathologists; how to discriminate different types of pathology in the same brain. This is particularly problematic when a brain harbors AD-type pathology and PSP-type pathology because both are characterized by tau deposits [3, 11]. Furthermore, it is not yet known whether these two types of pathology are independent or mutually related. In this study, we selected autopsy samples carrying histological diagnoses of both AD and PSP. We tried to extract disease-specific features from these brains with AD and PSP for possible discrimination, based on the distribution of TAs and that of NFTs and immunohistochemistry for phosphorylated tau (AT8) [24], three-repeat (3R) and four-repeat (4R) tau [9]. This hybrid approach was quite successful in discriminating most, but not all, of tau-positive lesions, suggesting that AD-type pathology and PSP-type pathology are independent without significant interaction even when coexistent in the same brain.

### Patients and methods

Among one hundred and eighty autopsy cases archived at Laboratory of Structural Neuropathology, Tokyo Metropolitan Institute of Medical Science from 1999 to 2013, we picked up 5 cases (3 cases from Nitobe Memorial Nakano General Hospital and 2 cases from Yokufukai hospital) with comorbid pathologies with PSP [15] and AD [5]. Written consent from the patient’s family was obtained at autopsy and this study was approved at the ethics review committee of the Tokyo Metropolitan Institute of Medical Science (authorization number 16–25).

Demographic data are summarized in Table 1. Median age was 85 years (range 80–94 years) and male female ratio was 4:1. Brain weight was 1260 g on average (range 1205–1395 g), Braak NFT stage [5] had median V (range II-VI), and Braak Amyloid stage [5] had median B (range 0 - C).

**Table 1:** Demographic data on 5 patients with neuropathological diagnosis of AD and PSP.

Case	Age at death	Sex	Clinical diagnosis	Dementia	Parkinsonism	Duration of illness (year)	Neuropathological findings	Brain weight (g)	Braak NFT stage	Braak Amyloid stage
1	86	F	CHF	–	–	N. A	PSP, AD	1205	II	0
2	94	M	possible DLB	+	+	3	LB pathology, PSP, AD, AGD	1260	III	A
3	81	M	AD	+	–	9	PSP, AD	1230	V	C
4	85	M	pneumonia	N. A	N. A	N. A	PSP, AD, CAA	1395	V	C
5	80	M	possible PSP	+	+	8	PSP, AD, AGD	1210	VI	C

*AD* Alzheimer disease, *AG* argyrophilic grain disease, *CAA* cerebral amyloid angiopathy, *CHF* congestive heart failure, *DLB* dementia with Lewy body, *N. A* not available, *NFT* neurofibrillary tangle, *PSP* progressive supranuclear palsy

Sixteen regions, selected to map PSP-specific or AD-specific lesions [2, 4–6, 14, 18, 22, 34, 40] throughout the brain include primary motor cortex (PC), putamen (PU), external segment of globus pallidus (GPE), internal segment of globus pallidus (GPI), subthalamic nucleus (STN), hippocampal formation (HF), substantia nigra (SN), red nucleus (RN), central grey matter (CGM), tegmentum of midbrain (mainly superior coliculli, excluding CGM;M-TEG), locus coeruleus (LC), raphe nucleus (RPN), pontine nucleus (PN), tegmentum of pons (excluding LC and RPN;P-TEG), inferior olivary nucleus (ION) and dentate nucleus (DN). Six micron-thick sections were obtained from the formalin-fixed, paraffin-embedded blocks from these 16 regions. Deparaffinized sections were subjected to Hematoxylin-Eosin (HE) stain, Klüver-Barrera (KB) stain, Gallyas silver impregnation and Campbell silver impregnation [36]. Isoform-specific antibodies directed against 3R or 4R tau [9] were used [37, 41]. Briefly, deparaffinized sections were treated for 15 min with 0.25% potassium permanganate (KMnO_4_), for 3 min with 2% oxalic acid (OA), for 30 min with > 99% formic acid (FA) and for 20 min autoclaved at 121 °C in 0.05 M citrate buffer [16, 38]. After intrinsic peroxidases were inactivated by 1% hydrogen peroxide (H_2_O_2_) for 15 min, sections were incubated first with 5% horse serum in 0.01 M phosphate-buffered saline containing 0.03% polyoxyethylene (10) octylephenyl ether (Triton X-100, Wako, Tokyo, Japan; PBST). They were then incubated with either 3R tau-specific antibody (RD3 1:3000, Merck Millipore, Germany) or 4R tau-specific antibody (RD4 1:1000, Merck Millipore, Germany) diluted in the same buffer for 2 days at 4 °C [9]. They were then incubated with biotinylated secondary antibody against mouse IgG (1:1000, ABC Elite, Vector, Burlingame, CA) diluted in the same buffer for 2 h at room temperature. They were then incubated with avidin-biotin-peroxidase complex (1:1000, ABC Elite, Vector) for 1 h and visualized with diaminobenzidine and nickel ammonium chloride [38]. 4R or 3R immunoreactivity (IR) in each of 16 regions was separately evaluated semiquatitatively as none: 0, mild (1 to 5): +; moderate (6 to 10): ++; severe (> 10): +++, (lesions/visual field with × 20 objective).

In some areas, where both 3R and 4R tau immunoreactivities (IR) were coexistent, double immunofluorolabeling was performed as described previously. The 6-μm thick sections were deparaffinized for double immunofluorolabeling with antibodies against isoform-specific anti-4R tau antibody (rabbit polyclonal, Cosmo Bio Co, Tokyo, Japan), raised against amino acids 275–291 of human 4R tau, which is deaminated at N279 [8], and the anti-3R tau antibody (RD3) [9]. Sections were washed with PBST, blocked for 30 min in 5% normal goat serum/0.05% sodium azide /PBS and incubated with the polyclonal anti-4R-tau antibody (1:3000) and RD3 (1:300), diluted in the blocking buffer at 4 °C for 4 days. To reduce autofluorescence of lipofuscin, sections were treated with Sudan Black B [28]. These primary antibodies were labeled with Alexa 488 conjugated with anti-rabbit IgG (Molecular Probes, Oregon, USA, 1:200) and Alexa 568 conjugated with anti-mouse IgG (Molecular Probes, Oregon, USA, 1:200), respectively, diluted in PBS with 0.03% Triton X-100 overnight in the dark. Sections were mounted with buffered glycerol containing 0.1%*p*-phenylenediamine. Fluorescent signals were separately captured on a confocal system (Leica SP8; Leica Microsystems GmbH, Heidelberg, Germany) through a 63 x objective (NA 1.45).

## Results

**Fig. 1:**
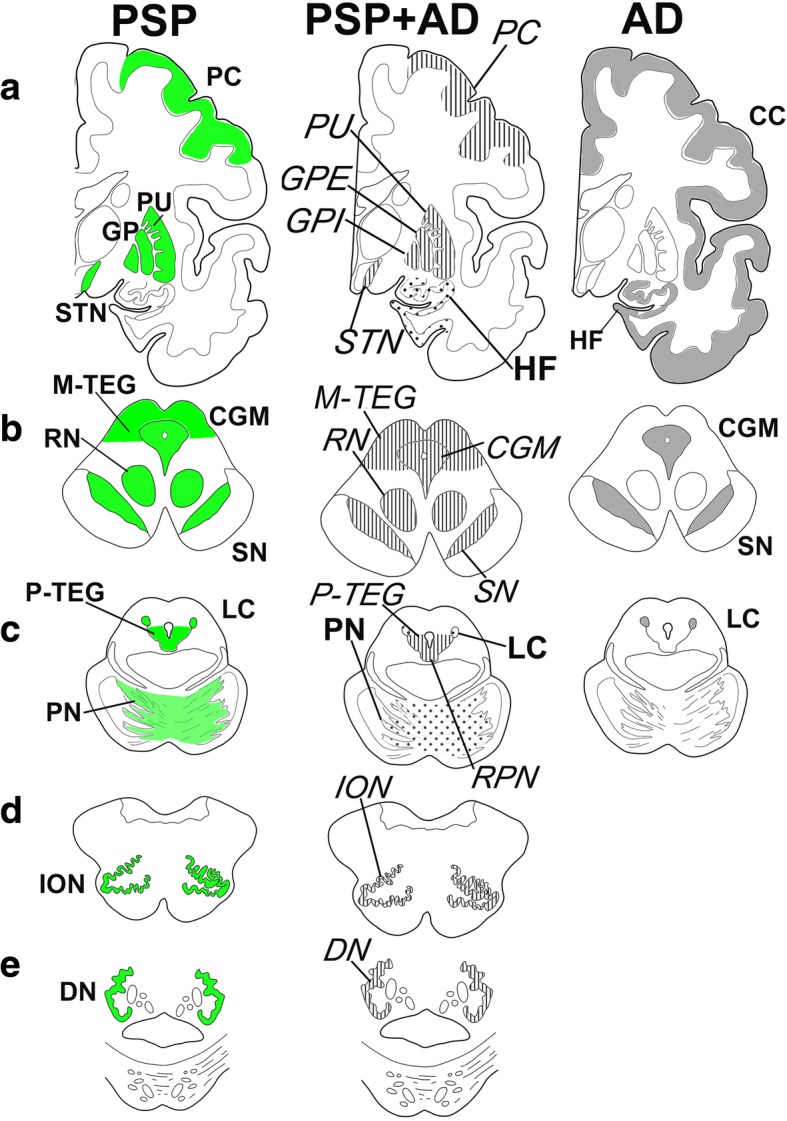
Separation of tau-positive lesions into progressive supranuclear palsy (PSP) and Alzheimer disease (AD) based on their distribution and cytopathology. Tau-positive lesions of PSP shown in green (left column, **a**-**e**), typically include primary motor cortex (PC), putamen (PU), globus pallidus (GP), subthalamic nucleus (STN), central grey matter (CGM), substantia nigra (SN), red nucleus (RN), tegmentum (M-TEG), locus coeruleus (LC), pontine nucleus (PN) and tegmentum (P-TEG), inferior olivary nucleus (ION) and dentate nucleus (DN) in the cerebellum. Tau-positive lesions of Alzheimer disease (AD), shown in grey (right column A-C), are more restricted to CGM, SN and LC in the brainstem while more extended in the hippocampal formation (HF) and cerebral cortex (CC). In the five comorbid cases with PSP and AD (PSP + AD in the mid column), these tau-positive lesions are partly overlapping. Regions with neurofibrillary tangles (NFTs) with tuft-shaped astrocytes (TAs) are labeled in italics, which replicate of PSP-type distribution (left column). Those with NFTs without TAs are labeled in Bold face (HF, LC and PN), which replicate AD-type distribution. Cerebral left hemisphere (coronal), **b**: Midbrain (axial), **c**: Pons (axial), **d**: Medulla oblongata (axial), **e**: Cerebellum.

Tau-positive lesions detected by AT8 were sorted into TA and NFT and semiquantitatively mapped in 16 regions as shown in Table 2. The distribution of tau-positive lesions in these 5 comorbid cases was schematized in Fig. 1 (center column). Comparison with that of PSP (Fig. 1, left column) and that of AD (Fig. 1, right column) demonstrated that the overall tau distribution in these five comorbid cases (Fig. 1, center column) was compatible with the summation of PSP (Fig. 1, left column) and AD (Fig. 1, right column). However, distribution of TA and that of NFT were not similar. Both TA and NFT were detected in RN, ION, DN, GPI, GPE and PU (Tab. 2, italics), where tau-positive lesions are rare in pure AD. These regions containing both TA and NFT replicated the distribution of pure PSP (Fig. 1, left column), suggesting that these PSP-like tau lesions are not influenced by the copresence of AD pathology (Fig. 1, right column). However, both TA and NFT were found in PC, M-TEG, CGM, P-TEG and SN, where tau-positive lesions were found in pure AD as well (Tab. 2, Fig. 1).

**Table 2:**
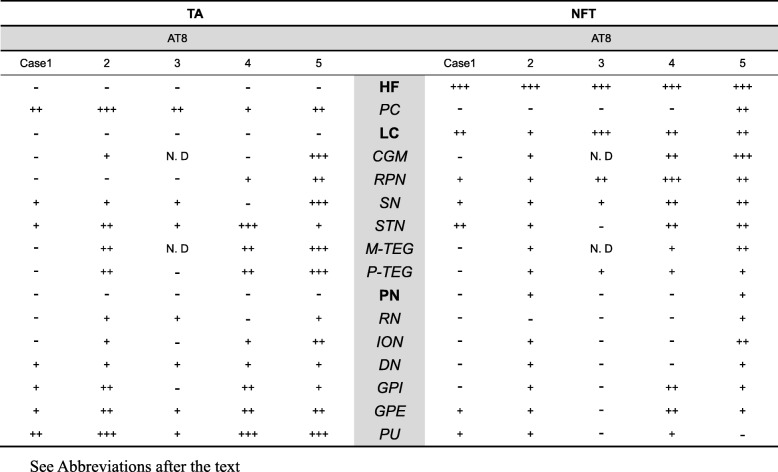
Regional distribution of AT8 positive lesions.

**Fig. 2:**
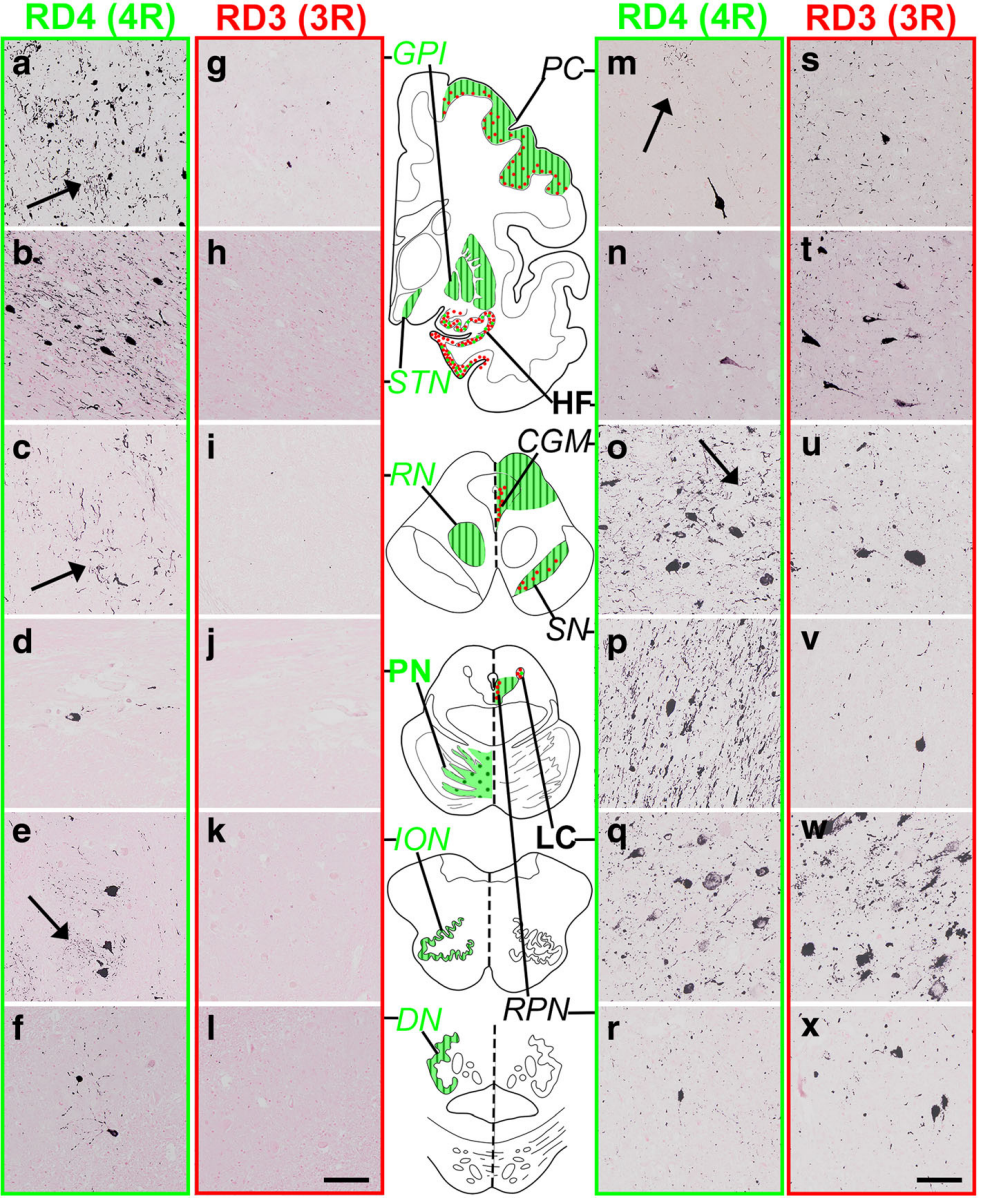
Tau isoform-oriented mapping into four repeat only (4R+/3R-) regions for PSP type and three and four repeat (3R+/4R+) regions for AD type distribution. Representative immunostaining for four-repeat (4R) tau (RD4, green) and three-repeat (3R) tau (RD3, red) in these twelve regions. The left set columns demonstrated 4R specific immunoreactivity (IR) without 3R IR in GPI (a, g), STN (b, h), RN (c, i), PN (d, j), ION (e, k) and DN (f, l), which replicates PSP-type distribution with glial involvement (arrows). In contrast, the right set columns demonstrated both 4R and 3R tau IR in PC (m, s), HF (n, t), CGM (o, u), SN (p, v), LC (q, w) and RPN (r, x), which replicates AD-type distribution. Glial lesions are exclusively positive for RD4 (arrows) but negative for RD3. Bars: 100 μm

To distinguish PSP-type and AD-type tau pathologies in these comorbid cases, tau-positive lesions, already sorted into NFT/TA (Tab. 2, Fig. 1), were further distinguished by 3R and 4R IR as shown with their relative amount in Table 3. Their immunohistochemical profiles are displayed in Fig. 2, where 4R-selective regions (Fig. 2, a-l, left: containing 4R+/3R- tau lesions) are contrasted with 4R + 3R regions (Fig. 2, m-x right: containing both 3R and 4R-positive lesions). TAs were positive only for 4R tau and found in every area examined except for LC, HF, PN. 3R tau-positive TAs were absent even when 3R-positive NFT pathology was prominent as in LC (Fig. 2w).

**Table 3:**
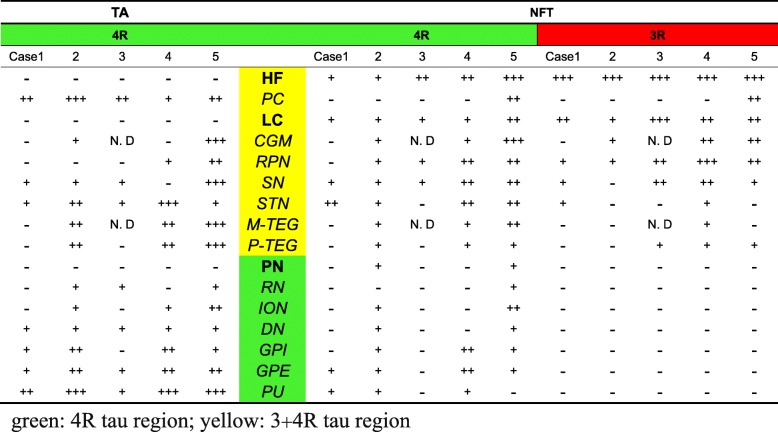
Regional distribution of 3R tau lesions and 4R tau lesions.

**Fig. 3:**
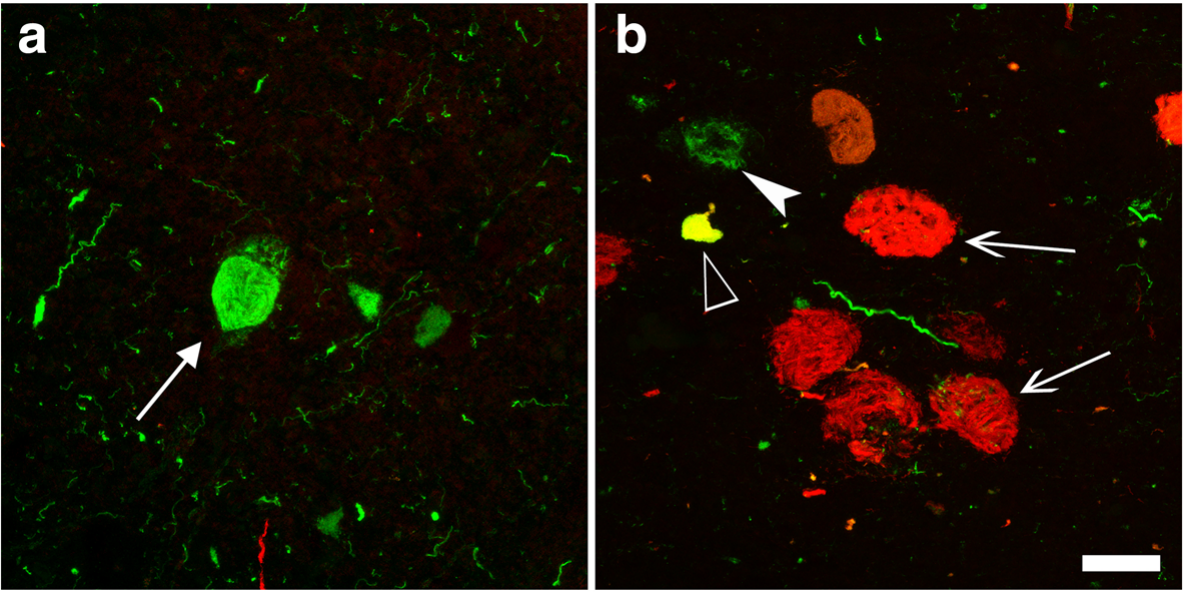
Tau isoforms on NFTs are different from a region (SN) to another (CGM) even in the same brain (Case 5). Preferential 4R labeling (green) on NFT (arrow) in SN (**a**) represents PSP-type pathology. In CGM (**b**), NFTs are differently labeled for 4R (green, arrowhead), 3R (red, arrows) or both 4R. and 3R (yellow, empty arrow), representing AD-type pathology. Bar: 25 μm

4R-selective regions were GPI, RN, PN, ION, DN (Fig. 2 a-l, Table 3, regions in green box), where NFTs were positive only for 4R (Fig. 2 a-l, left), while STN contained a few NFTs positive for 3R. In contrast, NFTs positive for 4R and 3R tau were found in 4R + 3R regions, including PC (Case 5 only), HF, CGM, SN, LC, RPN, M-TEG and P-TEG. (Fig. 2 m-x, Tab. 3, regions in yellow box). Among these 4R + 3R regions, HF and LC were characterized by dominant 3R IR in NFTs over 4R IR and lack of 4R-positive glia (Tab. 3). NFTs in PC of case 5 (Braak NFT stage VI) exhibited dominant 3R IR over 4R IR. In other 4R + 3R regions (CGM, RPN, SN, STN, M-TEG, P-TEG), 3R IR and 4R IR were comparable and tau-positive astrocytes were present in variable number. Double immunofluorolabeling (Fig. 3) for 4R tau (green) and 3R tau (red) demonstrated expected dominance of 4R tau (green) in 4R-selective regions as SN (Fig. 3a). In contrast, the proportion of 3R tau (red) and 4R tau (green) was variable from a neuron to another (Fig. 3b) in 4R + 3R regions as in CGM.

## Discussion

**Fig. 4:**
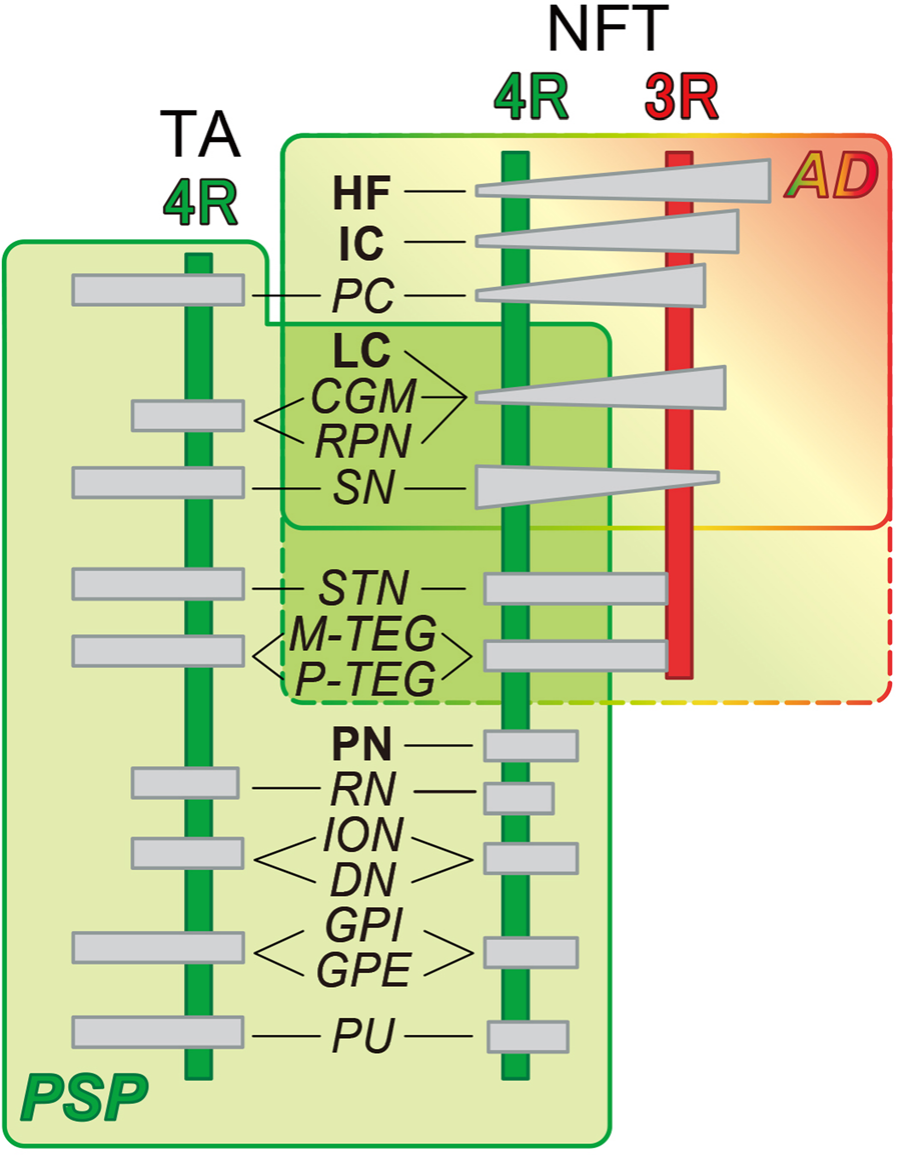
How to differentiate the origin (PSP or AD) of each tau-positive lesion in comorbid brains with both PSP and AD pathology. Tau-positive lesions are sorted along regions indicated at the center column. Regions containing NFTs but not TAs are labeled in bold face (HF, IC: insular cortex, LC and PN). Other regions containing both NFTs and TAs are labeled in italics. Horizontal bars indicate relative quantity of TA and NFT in each region. IR to 4R tau (green) and that to 3R tau (red) are indicated by vertical lines. TAs are positive only for 4R tau as indicated on the left half. In contrast, NFTs were sometimes positive for 3R tau in SN. 3R tau IR was more predominant in HF, IC, PC, LC, CGM and RPN, which replicates AD-like distribution partly shared with typical PSP. Rectangle in the right upper corner labeled as AD encompasses AD-type pathology in terms of distribution, further characterized by dominance of 3R tau and lack of TAs. The left lower area labeled as PSP encompasses PSP-type pathology in terms of distribution with 4R selectivity not only on TAs but also on NFTs. Although this chart provides concise and operational sorting of tau-positive lesions into AD or PSP origins, it is still difficult how to sort STN, M-TEG and P-TEG into PSP or AD (dotted area), because a few lesions exhibited 4R tau IR

Tau pathology of PSP-type and that of AD-type have been described separately [2, 4–6, 14, 22, 40], each representing separate entities. In this study with 5 cases harboring both PSP-type and AD-type tau pathology, we tried to discriminate PSP-type and AD-type pathologies in each brain along different criteria (distribution, participation of glial changes and tau isoforms). As initial description of PSP was based only on NFTs [31], comparison of their distribution provided a solid framework to distinguish pure PSP from pure AD (Fig. 1, Tab. 2). 4R-selective regions, characterized by the copresence of TA and NFT, both devoid of 3R tau IR (lower half of Tab. 3, in green box), include typical distribution of pure PSP (PN, RN, ION, DN, GPI, GPE and PU) [15], which are not severely affected in pure AD. In contrast, involvement of 3R tau is seen in HF and LC in this series of cases with both disorders, which strongly suggests AD-type pathology [17, 21, 33, 37] rather than PSP-pathology. This assumption is reinforced by the paucity of glial involvement in these regions as in pure AD cases [22] and by the predominance of 3R tau over 4R tau (Tab. 3) [12]. Therefore, neuron-selective involvement with preferential 3R tau over 4R tau may represent AD-type pathology [3, 23] even in this comorbid series, which replicated AD-type distribution of NFT (HF, IC, LC, Fig. 4, right upper rectangle labeled AD). This is in contrast with PSP-type pathology with 4R-selective tau in both TA and NFTs (Fig. 4, left lower area in green) [11, 15], which replicated the PSP-type distribution. This operational sorting through our hybrid approach was powerful enough to distinguish origin of most of tau lesions into either AD-type or PSP-type (Fig. 4), suggesting that AD-type pathology and PSP-type pathology are independent even when these two processes are occurring in the same brain [7, 18, 19, 25, 27].

This sharp distinction of AD-type pathology and PSP-type pathology in the same brain suggests that extension of AD-type pathology and PSP-type pathology are independently guided by distinct cytopathological mechanism along disease-specific patterns without crossover even if 4R tau is found in AD and PSP. If 4R-tau lesions extended transsynaptically (in a prion-like manner? [26]), AD-type and PSP-type lesions that are found together in some regions should also be found associated in their areas of projection. Because coexistent PSP-type pathology and AD-type pathology were distinct in the human brain, it is hard to explain how transsynaptic extension of 4R tau, for example if any, exhibit different types of tau pathology in the same brain. Still, there remain some ambiguities in some regions such as STN, SN, M-TEG and P-TEG (area in broken line, Fig. 4), where very small amount of 3R-poitive NFTs are sometimes present. In these regions, 3R-positive NFTs (arrows in Fig. 3b, red) may be of AD-type even they are positive also for 4R tau (empty arrowhead in Fig. 3b, yellow) [37, 39] . However, it is not yet clear whether 4R-positive neurons without 3R tau in these areas are of AD-type or PSP-type (Fig. 3b, arrowhead, green) [17, 37], because it is not yet possible to distinguish 4R tau of AD-type or of PSP-type. Indeed, 4R-tau lesions of AD and those of PSP similarly exhibit argyrophilia with Gallyas silver impregnation [36] and 4R tau IR with 4R-specific antibodies such as monoclonal antibody RD4 [9] or polyclonal antibodies to 4R tau [8].

It has been reported that pretangle neurons are positive for 4R tau but not for 3R tau in both AD and PSP [17, 37]. However, this selective 4R tau IR at early phase of tau deposition is gradually replaced with 3R tau IR during evolution NFTs in AD brain [12, 39]. This is in sharp contrast with PSP or CBD brain, where 4R tau IR remains persistent without involvement of 3R even after tau-positive fibrils are dense enough to form aggregated inclusions [20, 32]. Because currently available antibodies against 4R tau immunolabel tau deposits both in AD and in PSP/CBD brains [8], it remains to be clarified how 4R tau in AD brain and that in PSP brains are similar or different. However, if representation of 4R tau deposits is disease-specific, it is expected that molecular species of 4R tau itself is disease-specific as well. For example, it has been reported that asparagine at residue 279 of 4R tau is deamidated to aspartate in AD brains [13], while this posttranslational change is not robust in PSP/CBD brains [8]. Immunoprobes that may detect such AD-specific posttranslational changes may provide a straightforward strategy to demix PSP-type lesions and AD-type lesions in the same brain, in the same area or even within a single neuron at molecular level. If AD-type NFTs are characterized by paired helical filaments [29, 32] while PSP-type NFTs are characterized by straight fibrils [35] on electron microscopy, it will be exciting to examine how they are related to such biochemical differences, if any. Our hybrid approach to demix AD-type and PSP-type tau lesions may be corroborated by hybrid molecular demixing and electron microscopic studies, which will surely improve our mechanistic understanding of these diseases for more precise diagnosis and better management.

## Conclusions

In human autopsied brains harboring both PSP-type and AD-type pathologies, tau-positive lesions were sorted based on their cell type (neuron vs glia), distribution and tau isoforms (3R vs 4R). With this hybrid approach, we were successful in demixing PSP-type cytopathology (4R-selectivity in glia and neuron in PN, RN, ION, DN, GPI, GPE and PU) and AD-type cytopathology (3R and 4R in neuron in HF, insular cortex:IC and LC). However, this demixing is still incomplete because STN, SN, M-TEG and P-TEG contain tau lesions in neurons and glia are positive for 3R and 4R. Further demixing may be possible if biochemical difference of 4R tau between PSP and AD is identified.

## Abbreviations

3R: three-repeat
4R: four-repeat
AD: Alzheimer disease
CC: cerebral cortex
CGM: central grey matter
DN: dentate nucleus
FA: formic acid
GPE: external segment of globus pallidus
GPI: internal segment of globus pallidus
H_2_O_2_: hydrogen peroxide
HE: Hematoxylin-Eosin
HF: hippocampal formation
IC: insular cortex
ION: inferior olivary nucleus
IR: immunoreactivity
KB: Klüver-Barrera
KMnO_4_: potassium permanganate
LC: locus coeruleus
M-TEG: tegmentum of midbrain
NFT: neurofibrillary tangle
OA: oxalic acid
PBST: phosphate-buffered saline containing 0.03% polyoxyethylene (10) octylephenyl ether
PC: primary motor cortex
PN: pontine nucleus
PSP: progressive supranuclear palsy
P-TEG: tegmentum of pons
PU: putamen
RN: red nucleus
RPN: raphe nucleus
SN: substantia nigra
STN: subthalamic nucleus
TA: tuft-shaped astrocyte
